# Supplementary material for: Whole-Blood Gene Expression Profiles Associated with Mortality in Community-Acquired Pneumonia
Source: Biomedicines. 2023 Feb 1;11(2):429. doi: 10.3390/biomedicines11020429 (PMC9953679; doi:10.3390/biomedicines11020429)
Supplement: Supplementary file 1 [file biomedicines-11-00429-s001.zip › biomedicines-2110225-supplementary.pdf]

Supplementary material

Whole-blood gene expression profiles associated with mortality in community-acquired pneumonia

Diego Viasus, Antonella F. Simonetti, Lara Nonell, Oscar Vidal, Yolanda Meije, Lucía Ortega, Magdalena Arnal, Marta Bódalo-Torruella, Montserrat Sierra, Alexander Rombauts, Gabriela Abelenda-Alonso, Gemma Blanchart, Carlota Gudiol, Jordi Carratalà

Figure S1. Expression data for each gene and prognosis.

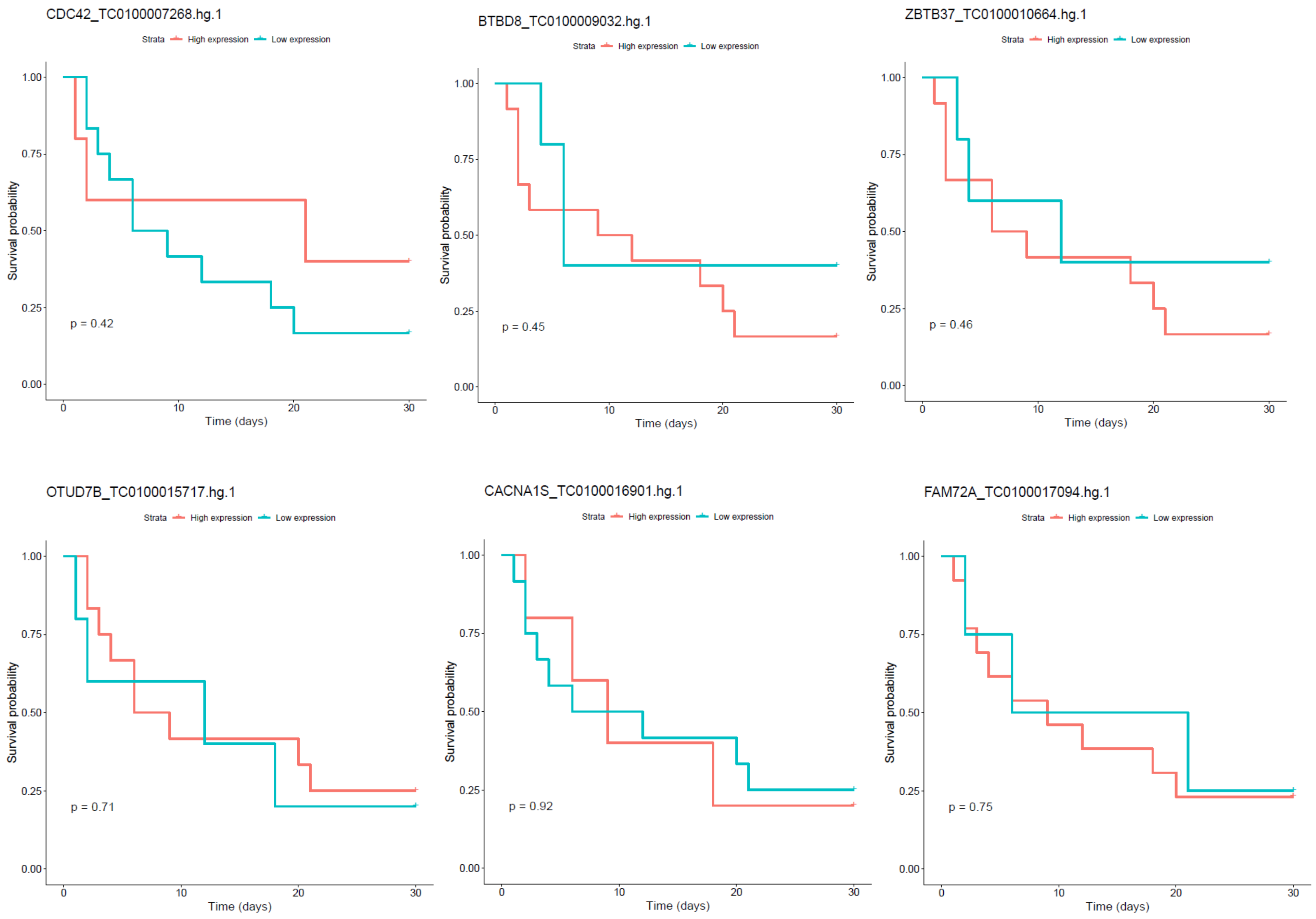

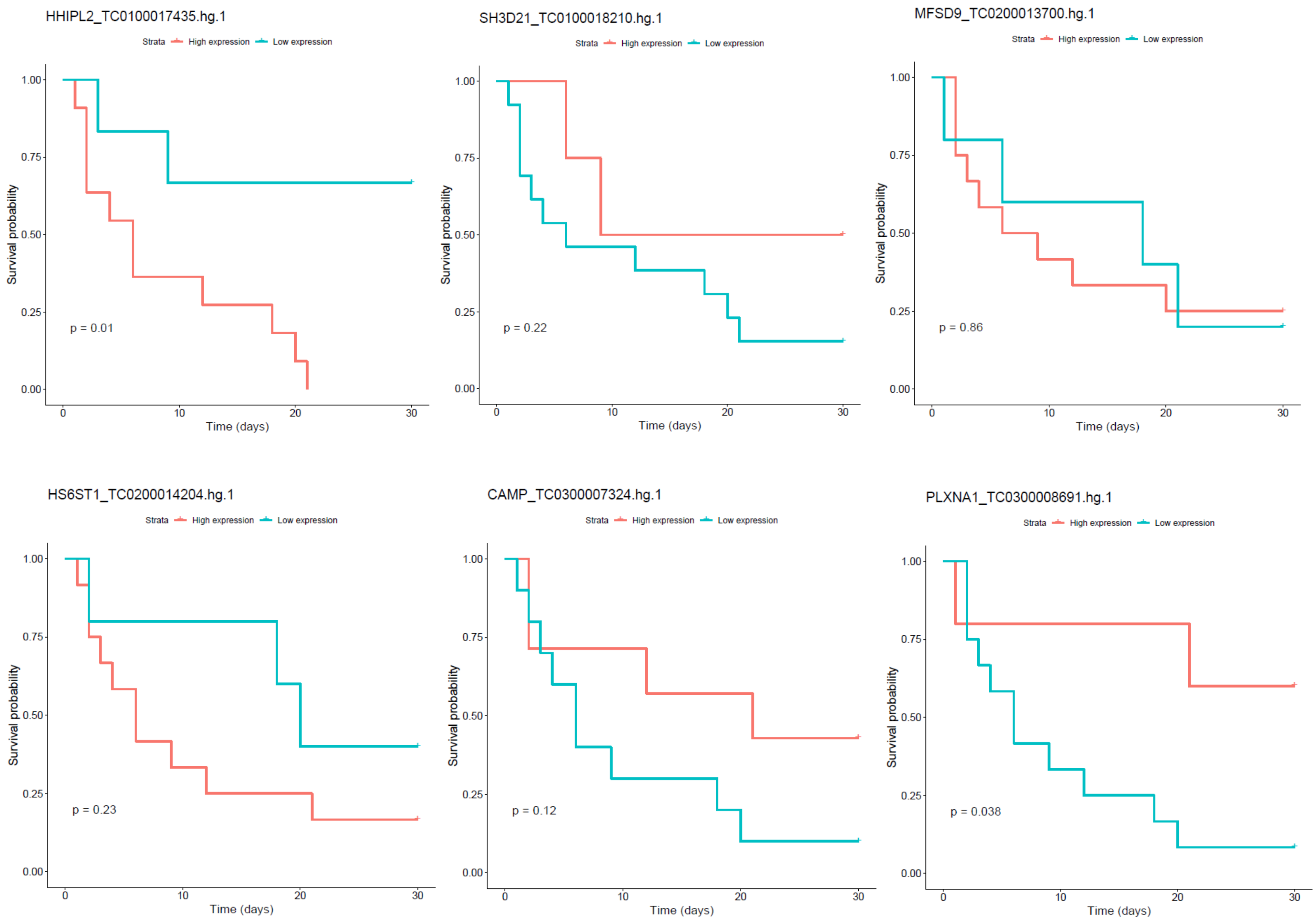
HHIPL2_TC0100017435.hg.1
Strata High expression Low expression
Survival probability
Time (days)
p = 0.01
SH3D21_TC0100018210.hg.1
Strata High expression Low expression
Survival probability
Time (days)
p = 0.22
MFSD9_TC0200013700.hg.1
Strata High expression Low expression
Survival probability
Time (days)
p = 0.86
HS6ST1_TC0200014204.hg.1
Strata High expression Low expression
Survival probability
Time (days)
p = 0.23
CAMP_TC0300007324.hg.1
Strata High expression Low expression
Survival probability
Time (days)
p = 0.12
PLXNA1_TC0300008691.hg.1
Strata High expression Low expression
Survival probability
Time (days)
p = 0.038

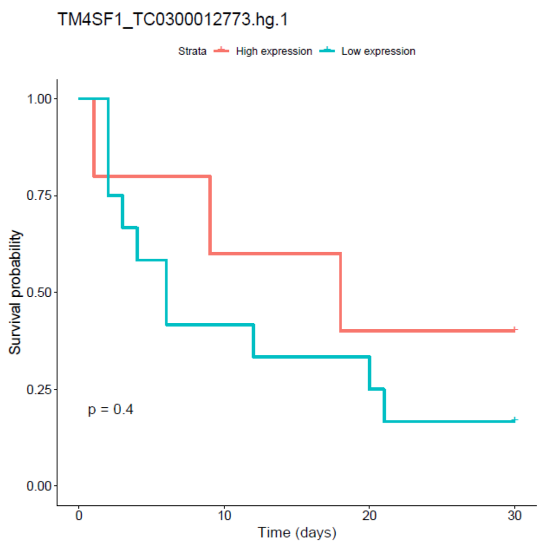
TM4SF1_TC0300012773.hg.1
Strata
High expression
Low expression
Survival probability
1.00
0.75
0.50
0.25
0.00
p = 0.4
0
10
20
30
Time (days)

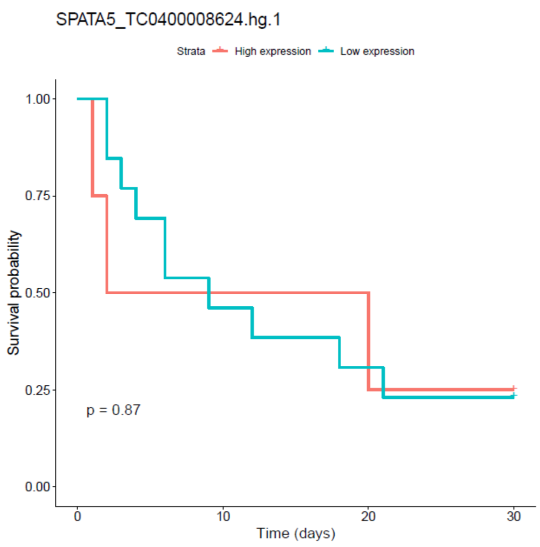
SPATA5_TC0400008624.hg.1
Strata
High expression
Low expression
Survival probability
1.00
0.75
0.50
0.25
0.00
p = 0.87
0
10
20
30
Time (days)

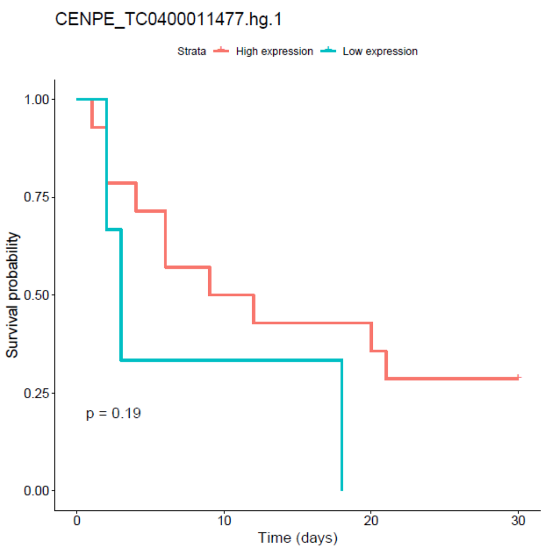
CENPE_TC0400011477.hg.1
Strata
High expression
Low expression
Survival probability
1.00
0.75
0.50
0.25
0.00
p = 0.19
0
10
20
30
Time (days)

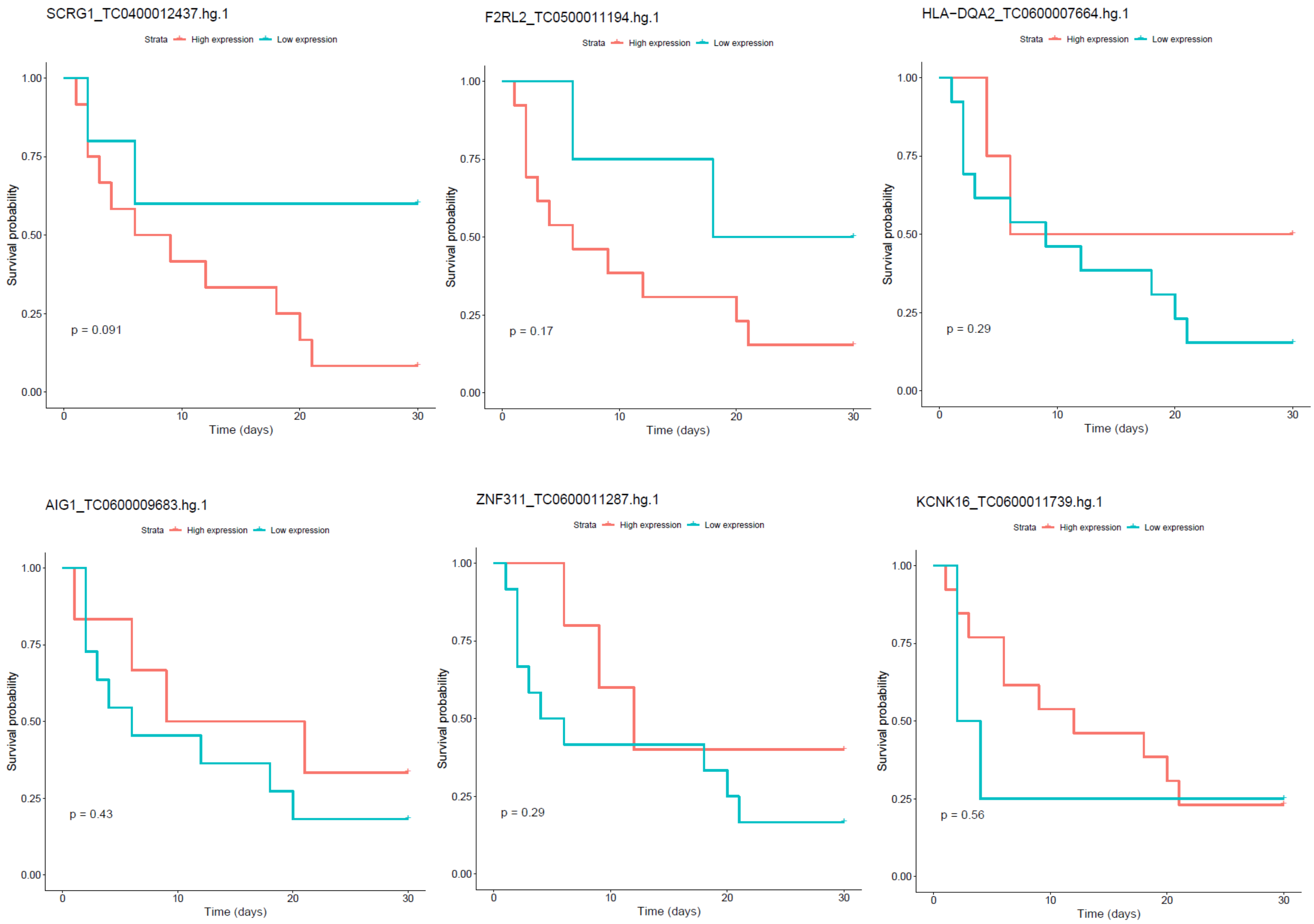
SCRG1_TC0400012437.hg.1
Strata
High expression
Low expression
Survival probability
1.00
0.75
0.50
0.25
0.00
p = 0.091
0
10
20
30
Time (days)
F2RL2_TC0500011194.hg.1
Strata
High expression
Low expression
Survival probability
1.00
0.75
0.50
0.25
0.00
p = 0.17
0
10
20
30
Time (days)
HLA−DQA2_TC0600007664.hg.1
Strata
High expression
Low expression
Survival probability
1.00
0.75
0.50
0.25
0.00
p = 0.29
0
10
20
30
Time (days)
AIG1_TC0600009683.hg.1
Strata
High expression
Low expression
Survival probability
1.00
0.75
0.50
0.25
0.00
p = 0.43
0
10
20
30
Time (days)
ZNF311_TC0600011287.hg.1
Strata
High expression
Low expression
Survival probability
1.00
0.75
0.50
0.25
0.00
p = 0.29
0
10
20
30
Time (days)
KCNK16_TC0600011739.hg.1
Strata
High expression
Low expression
Survival probability
1.00
0.75
0.50
0.25
0.00
p = 0.56
0
10
20
30
Time (days)

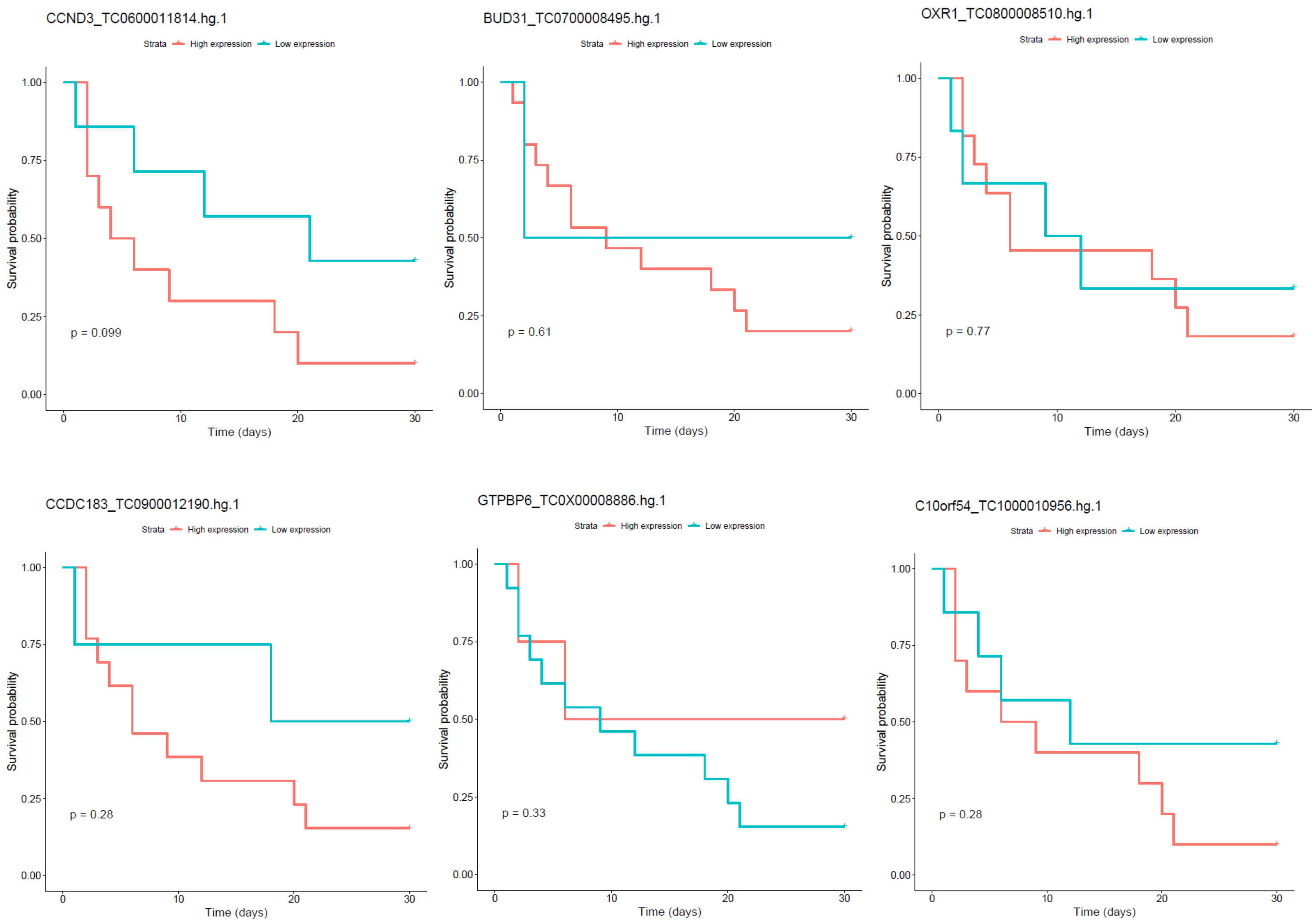
CCND3_TC0600011814.hg.1
Strata High expression Low expression
Survival probability
Time (days)
p = 0.099
BUD31_TC0700008495.hg.1
Strata High expression Low expression
Survival probability
Time (days)
p = 0.61
OXR1_TC0800008510.hg.1
Strata High expression Low expression
Survival probability
Time (days)
p = 0.77
CCDC183_TC0900012190.hg.1
Strata High expression Low expression
Survival probability
Time (days)
p = 0.28
GTPBP6_TC0X00008886.hg.1
Strata High expression Low expression
Survival probability
Time (days)
p = 0.33
C10orf54_TC1000010956.hg.1
Strata High expression Low expression
Survival probability
Time (days)
p = 0.28

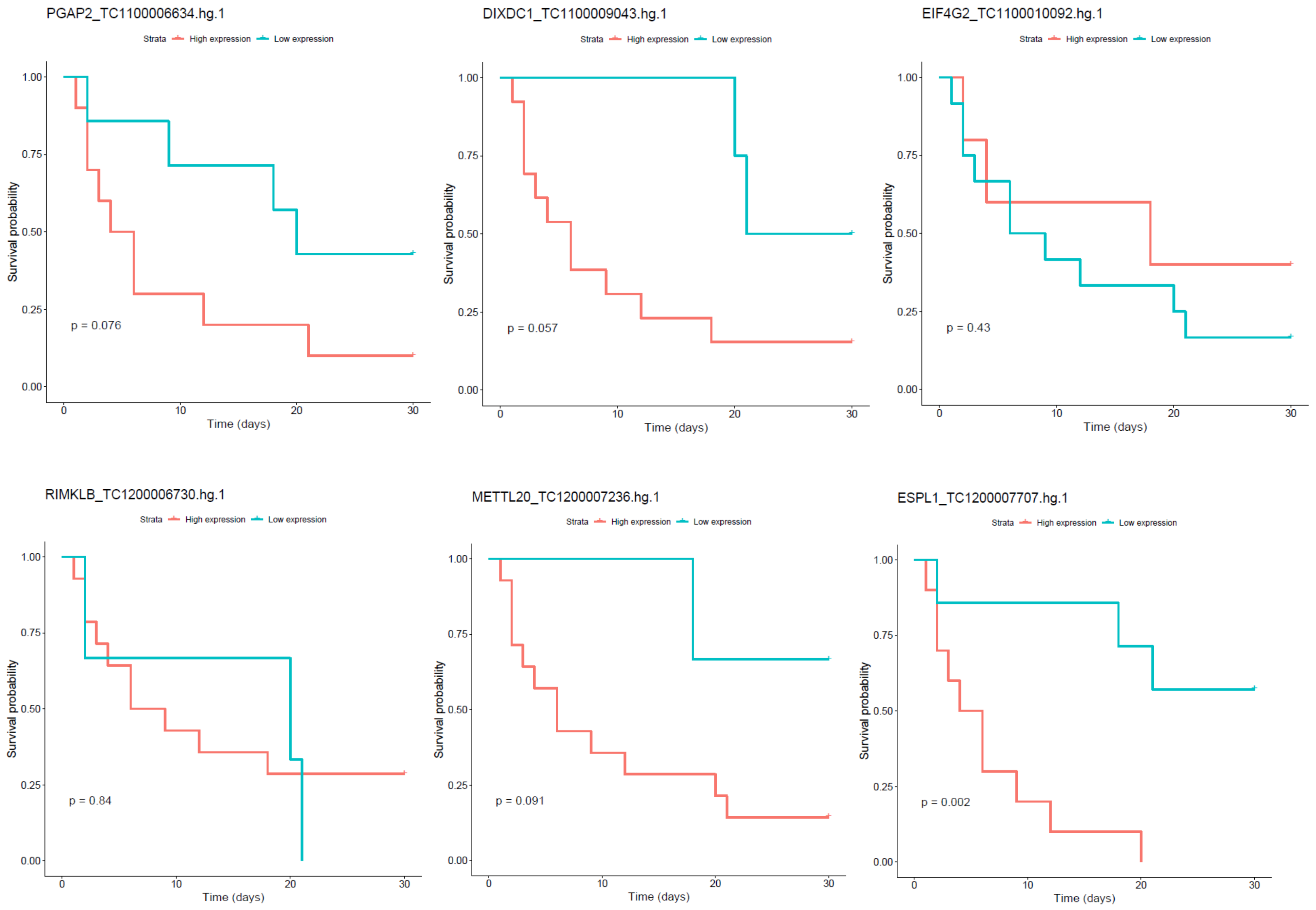
PGAP2_TC1100006634.hg.1
Strata
High expression
Low expression
Survival probability
Time (days)
p = 0.076
DIXDC1_TC1100009043.hg.1
p = 0.057
EIF4G2_TC1100010092.hg.1
p = 0.43
RIMKLB_TC1200006730.hg.1
p = 0.84
METTL20_TC1200007236.hg.1
p = 0.091
ESPL1_TC1200007707.hg.1
p = 0.002
0.00
0.25
0.50
0.75
1.00
0
10
20
30

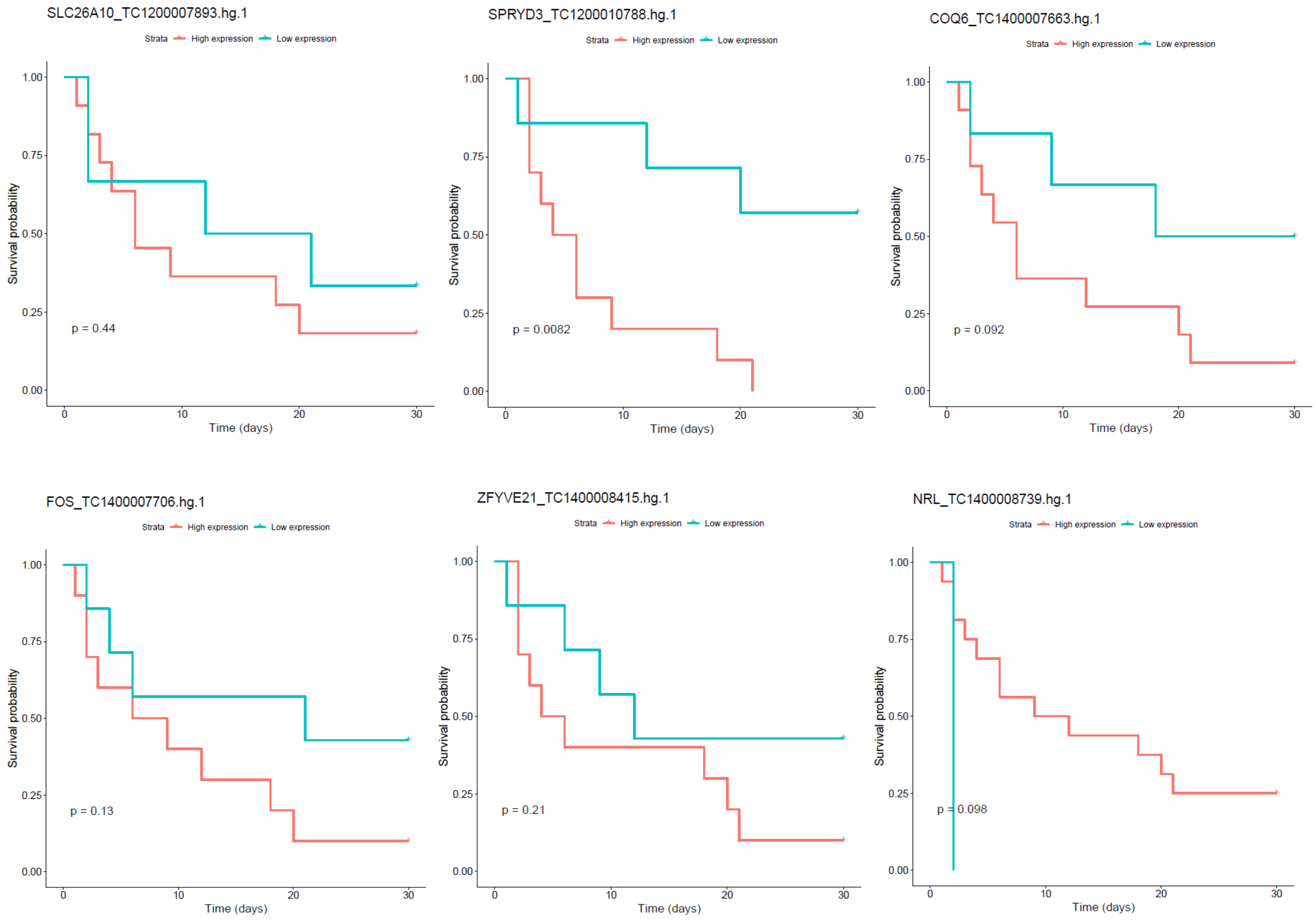

SLC26A10_TC1200007893.hg.1
Strata High expression Low expression
Survival probability
Time (days)
p = 0.44
SPRYD3_TC1200010788.hg.1
Strata High expression Low expression
Survival probability
Time (days)
p = 0.0082
COQ6_TC1400007663.hg.1
Strata High expression Low expression
Survival probability
Time (days)
p = 0.092
FOS_TC1400007706.hg.1
Strata High expression Low expression
Survival probability
Time (days)
p = 0.13
ZFYVE21_TC1400008415.hg.1
Strata High expression Low expression
Survival probability
Time (days)
p = 0.21
NRL_TC1400008739.hg.1
Strata High expression Low expression
Survival probability
Time (days)
p = 0.098

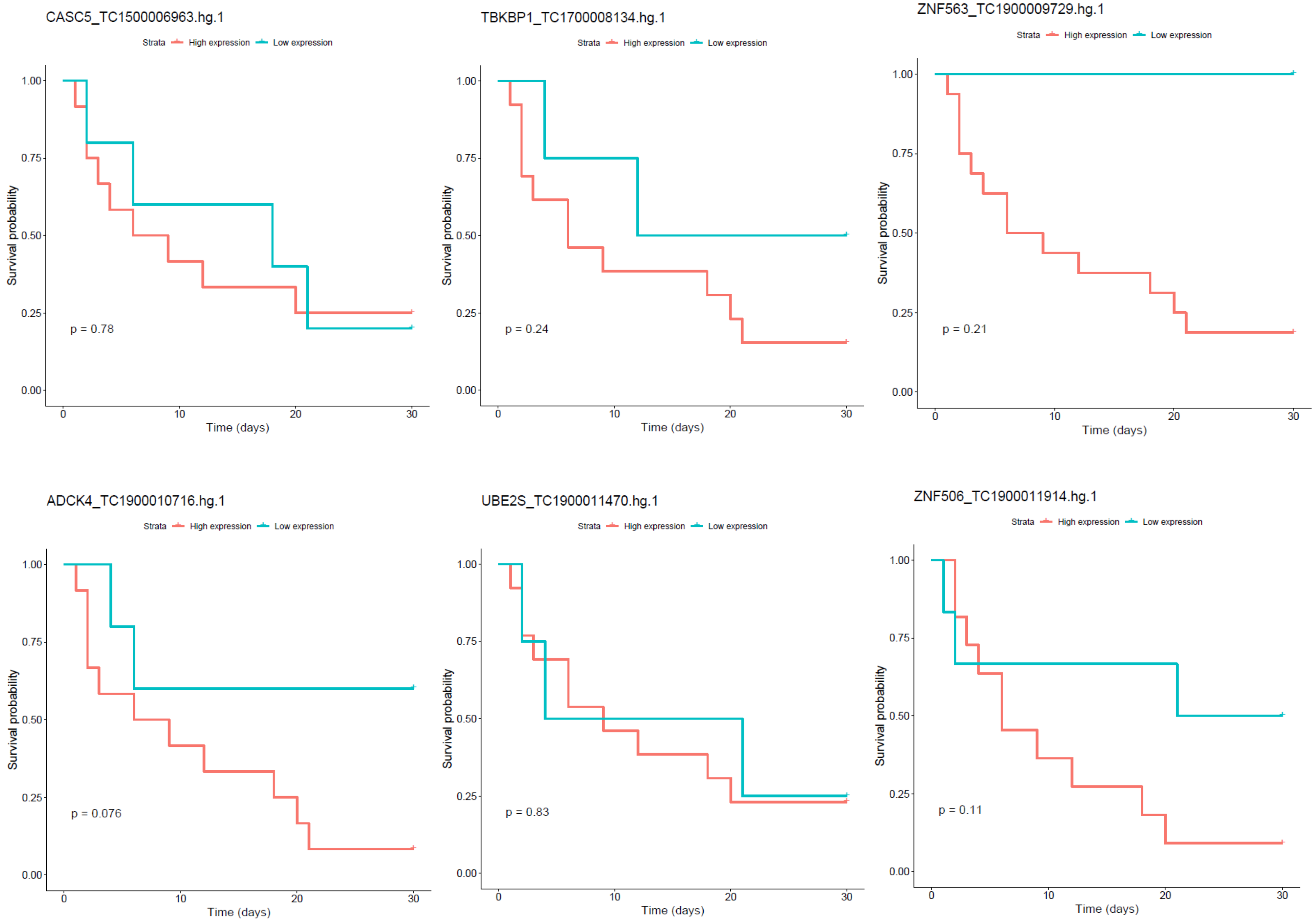

CASC5_TC1500006963.hg.1
Strata High expression Low expression
Survival probability
Time (days)
p = 0.78
TBKBP1_TC1700008134.hg.1
Strata High expression Low expression
Survival probability
Time (days)
p = 0.24
ZNF563_TC1900009729.hg.1
Strata High expression Low expression
Survival probability
Time (days)
p = 0.21
ADCK4_TC1900010716.hg.1
Strata High expression Low expression
Survival probability
Time (days)
p = 0.076
UBE2S_TC1900011470.hg.1
Strata High expression Low expression
Survival probability
Time (days)
p = 0.83
ZNF506_TC1900011914.hg.1
Strata High expression Low expression
Survival probability
Time (days)
p = 0.11

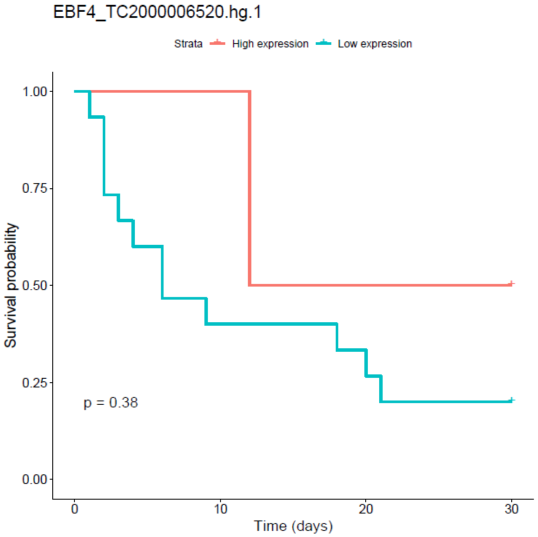
EBF4_TC2000006520.hg.1
Strata
High expression
Low expression
Survival probability
1.00
0.75
0.50
0.25
0.00
p = 0.38
0
10
20
30
Time (days)

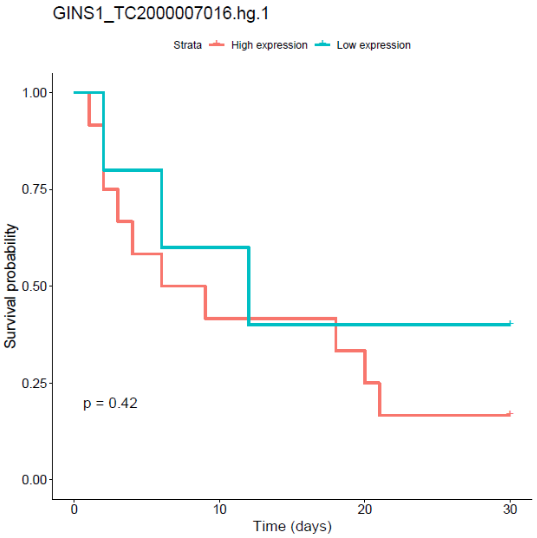
GINS1_TC2000007016.hg.1
Strata
High expression
Low expression
Survival probability
1.00
0.75
0.50
0.25
0.00
p = 0.42
0
10
20
30
Time (days)

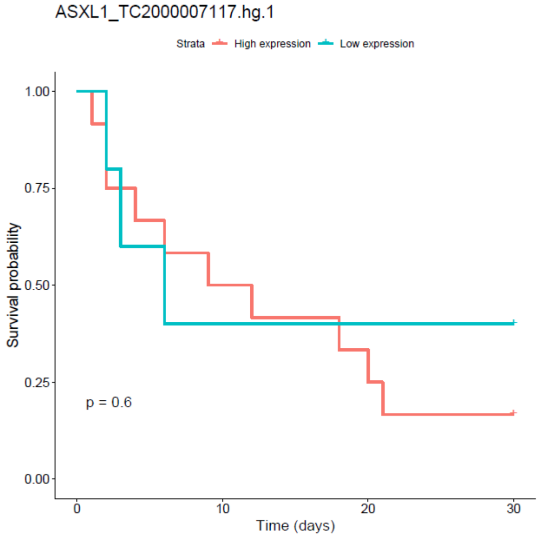
ASXL1_TC2000007117.hg.1
Strata
High expression
Low expression
Survival probability
1.00
0.75
0.50
0.25
0.00
p = 0.6
0
10
20
30
Time (days)

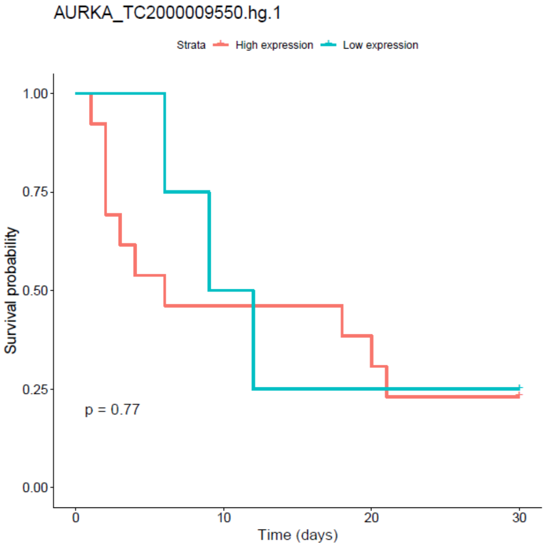
AURKA_TC2000009550.hg.1
Strata
High expression
Low expression
Survival probability
1.00
0.75
0.50
0.25
0.00
p = 0.77
0
10
20
30
Time (days)

Figure S2. Enrichment plot of gene sets.

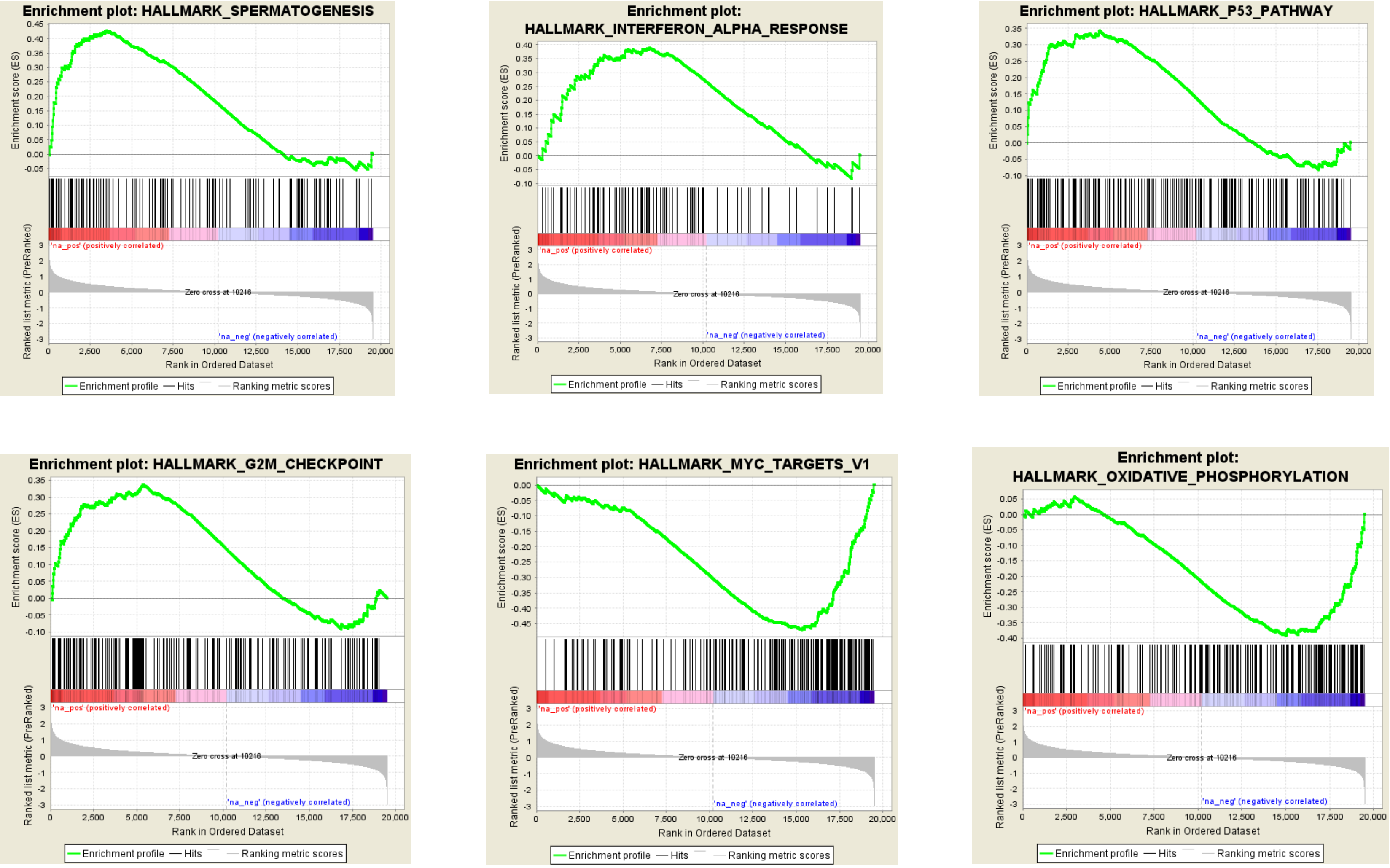

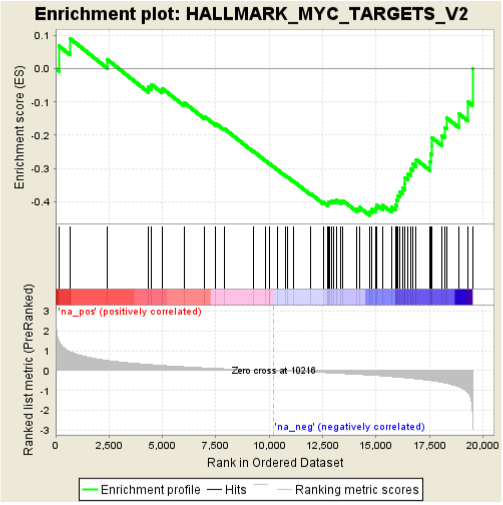
Enrichment plot: HALLMARK_MYC_TARGETS_V2
Enrichment score (ES)
Ranked list metric (PreRanked)
'na_pos' (positively correlated)
Zero cross at 10216
'na_neg' (negatively correlated)
Rank in Ordered Dataset
Enrichment profile
Hits
Ranking metric scores

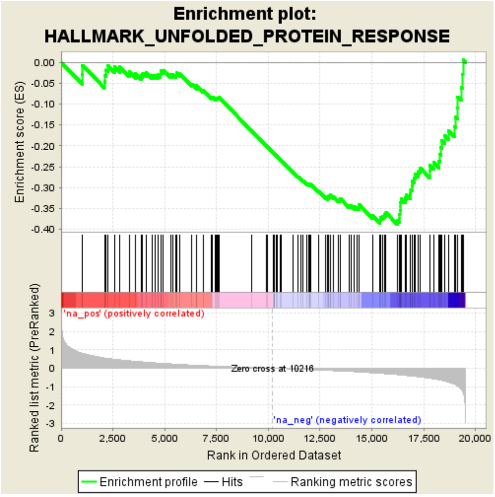
Enrichment plot:
HALLMARK_UNFOLDED_PROTEIN_RESPONSE
Enrichment score (ES)
Ranked list metric (PreRanked)
'na_pos' (positively correlated)
Zero cross at 10216
'na_neg' (negatively correlated)
Rank in Ordered Dataset
Enrichment profile
Hits
Ranking metric scores

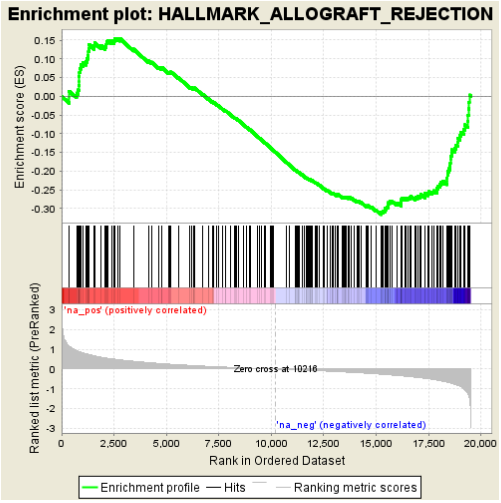
Enrichment plot: HALLMARK_ALLOGRAFT_REJECTION
Enrichment score (ES)
Ranked list metric (PreRanked)
'na_pos' (positively correlated)
Zero cross at 10216
'na_neg' (negatively correlated)
Rank in Ordered Dataset
Enrichment profile
Hits
Ranking metric scores

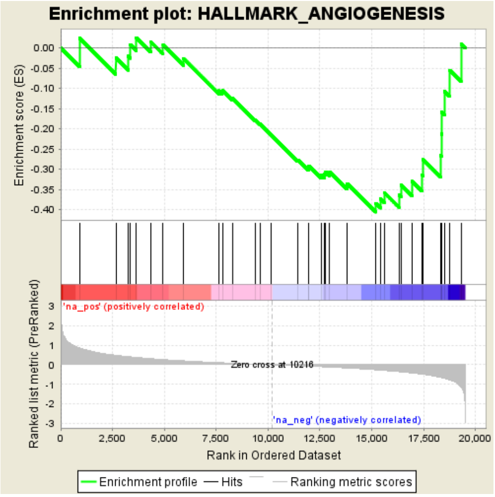
Enrichment plot: HALLMARK_ANGIOGENESIS
Enrichment score (ES)
Ranked list metric (PreRanked)
'na_pos' (positively correlated)
Zero cross at 10216
'na_neg' (negatively correlated)
Rank in Ordered Dataset
Enrichment profile
Hits
Ranking metric scores

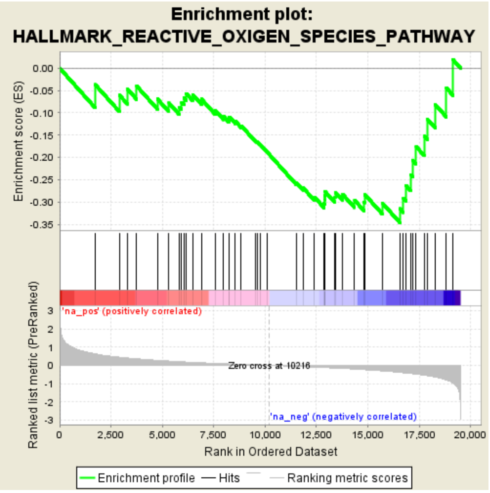
Enrichment plot:
HALLMARK_REACTIVE_OXIGEN_SPECIES_PATHWAY
Enrichment score (ES)
Ranked list metric (PreRanked)
'na_pos' (positively correlated)
Zero cross at 10216
'na_neg' (negatively correlated)
Rank in Ordered Dataset
Enrichment profile
Hits
Ranking metric scores
